# Knoevenagel Reaction in [MMIm][MSO_4_]: Synthesis of Coumarins

**DOI:** 10.3390/molecules16064379

**Published:** 2011-05-27

**Authors:** Pedro Verdía, Francisco Santamarta, Emilia Tojo

**Affiliations:** Department of Organic Chemistry, Faculty of Chemistry, University of Vigo, 36310 Vigo, Spain; Email: pedroverdia@uvigo.es (P.V.); bor164@uvigo.es (F.S.)

**Keywords:** ionic liquid, Knoevenagel reaction, synthesis, coumarins

## Abstract

The ionic liquid 1,3-dimethylimidazolium methyl sulfate, [MMIm][MSO_4_], together with a small amount of water (the amount taken up by the ionic liquid upon exposure to air), acts efficiently as both solvent and catalyst of the Knoevenagel condensation reactions of malononitrile with 4-substituted benzaldehydes, without the need for any other solvent or promoter, affording yields of 92%–99% within 2–7 min at room temperature. When L-proline is used as an additional promoter to obtain coumarins from *o*-hydroxybenzaldehydes, the reaction also proceeds in high yields. Work-up is very simple and the ionic liquid can be reused several times. Some of the coumarins obtained are described for the first time.

## 1. Introduction

The Knoevenagel condensation [1] is one of the most useful C=C bond forming reactions in organic synthesis [2]. The α,β-unsaturated products obtained have been widely used as intermediates in the synthesis of therapeutic drugs [3], natural products [4], functional polymers [5], fine chemicals [6], herbicides and insecticides. It is generally performed in organic solvents and catalysed by organic bases such as piperidine or pyridine [2]. Many of this conditions are associated with disadvantages such as hazardous and carcinogenic solvents and unrecoverability of the catalysts, which limit the use of these reactions in industrial processes.

Over the past decade, ionic liquids (ILs) have attracted great interest because of their unusual properties, including negligible vapour pressure, broad liquid temperature range, and high capacity as specific solvents [7]. Their use in place of hazardous volatile organic solvents makes organic synthesis environmentally benign [8]. In recent years they have been used in a number of Knoevenagel reactions as solvents and/or catalysts with considerable success [9,10,11], often accelerating the reaction, making work-up easier, and allowing their recycling [12,13,14]. In this work we have found that [MMIm][MSO_4_] containing a small amount of water, without any other promoter, can act efficiently as the reaction medium and catalyst of the Knoevenagel condensation of 4-substituted benzaldehydes. We report here the results of optimizing the amount of water in the medium and of applying this reaction to the synthesis of coumarins.

## 2. Results and Discussion

### 2.1. Knoevenagel Condensation of Benzaldehyde and Malononitrile

**Scheme 1:**
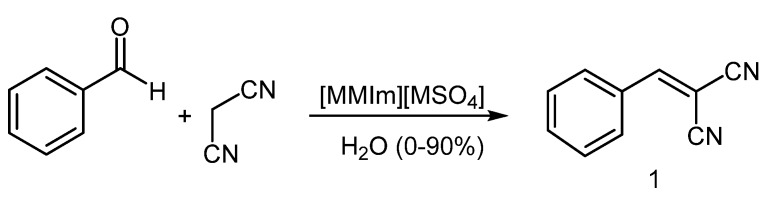
Knoevenagel condensations in undried [MMIm][MSO_4_]/H_2_O.

In direct continuation of our earlier research [15], we investigated whether anhydrous [MMIm][MSO_4_] could act as both solvent and catalyst (Scheme 1). The IL was prepared and characterized by ^1^H-NMR and FABMS, as we described in a previous paper [16]. The experimental procedure for the Knoevenagel reaction was very simple: A mixture of benzaldehyde, malononitrile and anhydrous IL was stirred at r.t., and the product was extracted from the reaction medium with ethyl acetate. The result is shown in Table 1, run 1.

**Table 1:** Effect of water on the Knoevenagel reaction in [MMIm] [MSO_4_].

Run ^a^	Time[min]	Yield [%] ^b^	% H_2_O
1	1080	40	-
2	2	99	2
3	2	98	5
4	2	97	10
5	8	93	15
6	10	86	25
7	11	83	50
8	300	81	90

*^a^* All runs were conducted at r.t.; *^b^* Yields refer to pure isolated product.

Since water can significantly influence the physicochemical properties of ionic liquids, the reaction was repeated using [MMIm][MSO_4_] and 25% water. Gratifyingly, the reaction time decreased to 10 min and the yield increased to 86%. We accordingly undertook to optimize the proportions of IL and water. As Table 1 shows, best results (2 min, 99%) were obtained with 2% of water; water contents higher than 10% led to increasingly longer reaction times and lower yields. 2-(Phenylmethylene) malononitrile (**1**) was obtained as a pure white solid that was identified by comparison of its physical and spectroscopic data with those reported in the literature [17].

The effect of water in these reactions is attributable in part to its lubricating action, *i.e.*, to its effect in reducing viscosity by decreasing the coulombic interactions between cation and anion in the IL [18], thereby allowing the ions greater translational and rotational freedom. Furthermore, the decrease in coulombic interactions is largely due to hydrogen bonding with anions [19,20] that should favor the catalytic effect of the IL, which is attributable to hydrogen-bonding between the imidazolium H-2 atom and the carbonyl group of the benzaldehyde [21,22].

Knowing that [MMIm][MSO_4_] absorbs 2.16% of water when exposed to the atmosphere (water content was determined using a Karl Fischer 756 coulometer), we then used the IL without drying and without adding either water or any other promoter. To evaluate the influence of recycling on the efficiency of undried [MMIm][MSO_4_], the ethyl acetate used to extract the reaction product was evaporated under vacuum and the medium was reused for the same reaction. After a total of six such cycles, yield was still high (96%), though the reaction time had increased to 4 h (Table 2).

**Table 2:** Reused of undried [MMIm][MSO_4_] (2.16% H_2_O) in successive reaction cycles.

Cycle ^a^	Time[min]	Yield [%] ^b^
1	2	99
2	12	99
3	19	99
4	50	98
5	70	99
6	240	96

*^a^* All runs were conducted at r.t.; *^b^* Yields referred to pure isolated product.

### 2.2. Knoevenagel Condensation with Different Substrates

**Scheme 2:**
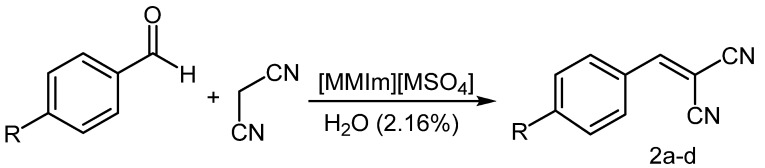
Knoevenagel condensations in undried [MMIm][MSO_4_].

To investigate the scope of the reaction by using undried [MMIm][MSO_4_] as solvent and catalyst, it was carried out using variously substituted benzaldehydes to obtain compounds **2a–d** (Scheme 2, Table 3). Products with spectral and physical data in agreement with previously reported values were obtained in yields of 92%–99% and in reaction times that ranged from 3 to 7 min.

### 2.3. Synthesis of Coumarins

**Scheme 3:**
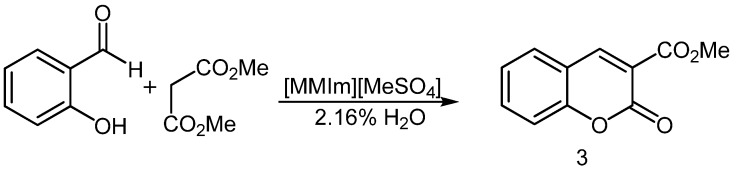
Synthesis of 3-(methoxycarbonyl)coumarin (**3**) in undried [MMIm][MSO_4_].

To apply the process to the synthesis of coumarins, we first tried to condense *o*-hydroxybenzaldehyde with dimethyl malonate in undried [MMIm][MSO_4_], with no other promoter, to obtain 3-(methoxy-carbonyl)coumarin (**3**, Scheme 3). However, after 18 h at 90 °C, no product had been formed (Table 4, run 1). By contrast, when the reaction was run in undried IL with L-proline as promoter, compound **3** could be extracted with ethyl acetate as a white solid that was positively identified by comparison of its physical and spectroscopic data with those reported in the literature [24]. Best results were obtained using 1 equiv. of L-proline at 90 °C, which afforded a yield of 98% in 30 min (Table 4).

**Table 3:** Knoevenagel condensations in undried [MMIm][MSO_4_] (2.16% H_2_O) as solvent and catalyst ^a^.

Entry	R	Time [min]	Yield [%]	Product	References
1	Cl	5	99	2a	[9]
2	NMe_2_	7	98	2b	[23]
3	OMe	3	92	2c	[9]
4	Me	5	98	2d	[23]

*^a^* All reactions were performed at r.t. following the same procedure as for the reaction of benzaldehyde with malononitrile.

**Table 4:** Synthesis of 3-(methoxycarbonyl)coumarin (**3**) in undried [MMIm][MSO_4_] with or without L-proline as promoter.

Run	L-proline [equiv.]	T [°C]	Time [h]	Yield [%]
1	-	90	18	-
2	0.2	90	4.5	96
3	0.5	90	1	90
4	1	90	0.5	98
5	1	70	2	90
6	1	50	6	80

**Scheme 4:**
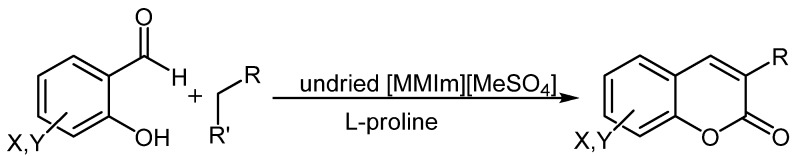
Synthesis of coumarins in undried [MMIm][MSO4] containing L-proline.

A wide range of aromatic *o*-hydroxybenzaldehydes underwent condensations with a variety of active methylene compounds by this procedure to provide the corresponding coumarins as pure solids in one-pot and high yields (Scheme 4, Table 5).

Their structures were characterized by IR, ^1^H-NMR, ^13^C-NMR and MS and confirmed by comparison of its physical and spectroscopic data when those where previously reported. All the reactions were in general fast and clean. The results are summarized in Table 5. The coumarins obtained in entries 5, 7, 11, 13, 14 and 15 have not been previously described. Subsequent desulfonylation of coumarins 14, 15 and 17 will allow obtaining a series of coumarins present in sesquiterpenes with very important pharmacological properties, such as galvanic acid [25].

**Table 5:** Synthesis of coumarins in undried [MMIm][MSO_4_] containing L-proline (1 equiv.) ^a^.

Entry	X	Y	R	R’	Time [min]	Yield [%] ^b^	References
1	H	H	CO_2_Me	CO_2_Me	30	98	[24]
2	H	H	COMe	CO_2_Me	15	99	[26,27]
3	H	H	CO_2_Et	CO_2_Et	80	98	[28,29]
4	H	H	COPh	CO_2_Et	360	97	[30]
5	6-CH_3_	H	CO_2_Me	CO_2_Me	90	87	-
6	6-CH_3_	H	CO_2_Et	CO_2_Et	30	88	[31]
7	8-CH_3_	H	CO_2_Me	CO_2_Me	70	94	-
8	6-OMe	H	CO_2_Me	CO_2_Me	15	87	[32,33]
9	7-OMe	H	CO_2_Me	CO_2_Me	70	90	[29]
10	8-OMe	H	CO_2_Me	CO_2_Me	30	94	[32]
11	6-Br	8-OMe	CO_2_Me	CO_2_Me	15	99	-
12	5,6-benzo	H	CO_2_Me	CO_2_Me	90	99	[34]
13	H	H	SO_2_Me	CO_2_Et	90	96	-
14	7-OH	H	SO_2_Me	CO_2_Et	480	99	-
15	7-Br	H	SO_2_Me	CO_2_Et	360	93	-
16	H	H	SO_2_Ph	CO_2_Me	1440	92	[35,36]
17	7-OH	H	SO_2_Ph	CO_2_Me	1140	99	[35]

*^a^* All runs were conducted at 90 °C; *^b^* Yields refer to pure isolated products.

Evaluation of recycling performance showed that the mixture of undried [MMIm][MSO_4_] and L-proline can be reused at least four times without loss of yield, though the reaction time increased to 6 h (Table 6).

An attractive feature of the methods described above is that [MMIm][MSO_4_] is easy to prepare; is one of the least expensive ILs; has desirable physical properties such as good thermal stability and low viscosity; and, unlike some other ILs, has low cytotoxicity [37].

**Table 6:** Reuse of undried [MMIm][MSO_4_] + L-proline (1 equiv.) in successive cycles of 3-(methoxycarbonyl)coumarin (**3**) synthesis.

Run ^a^	Time [h]	Yield [%] ^b^
1	0.5	98
2	1	98
3	3	98
4	5	98
5	6	98

*^a^* All runs were conducted at 90 °C; *^b^* Yields refer to pure isolated products.

## 3. Experimental

### 3.1. General

Reagents were purchased from Aldrich and used as received. IR spectra were recorded on a MIDAC Prospect FT-IR spectrophotometer. NMR spectra were recorded on a Brucker ARX 400 with chemical shifts given in ppm and coupling constants in Hertz. Electrospray MS were recorded on a micrOTOF FOCUS spectrometer. Melting points were measured with a Gallenkamp apparatus.

### 3.2. General Procedure for Knoevenagel Condensation of Benzaldehyde with Malononitrile

A mixture of benzaldehyde (0.065 g, 0.61 mmol) and malononitrile (0.040 g, 0.61 mmol) in [MMIm][MSO_4_] + water (2 mL) was stirred in a round-bottomed flask until completion of the reaction as indicated by TLC (eluent: 4:1 hexane:AcOEt). The reaction mixture was then extracted with ethyl acetate (3 × 2 mL), and the pooled organic extract was concentrated under vacuum, affording a pure solid.

### 3.3. Recycling of Undried [MMIm][MSO_4_] (2.16% H_2_O) in the Knoevenagel Reaction of Benzaldehyde with Malononitrile

Reaction and work-up as above afforded a solid that required no further purification. The reaction medium was recovered by evaporation of ethyl acetate under vacuum, and was reused.

### 3.4. General Procedure for the Synthesis of Coumarins

A mixture of salicylaldehyde (0.146 g, 1.2 mmol), dimethyl malonate (0.158 g, 1.2 mmol) and L-proline (0.156 g, 1.2 mmol) in undried [MMIm][MSO_4_] (2 mL) was stirred in a round-bottomed flask until completion of the reaction as indicated by TLC (eluent: 8:2 hexane:AcOEt). The reaction mixture was then extracted with ethyl acetate (3 × 2 mL), and the pooled organic extract was concentrated under vacuum, affording a pure solid.

### 3.5. Recycling of Undried [MMIm][MSO_4_] (2.16% H_2_O) + L-Proline in the the Synthesis of 3-(Methoxycarbonyl)coumarin (entry 1, Table 4)

Reaction and work-up as above afforded a solid that required no further purification. The reaction medium was recovered by evaporation of ethyl acetate under vacuum, and was reused.

*3-(Methoxycarbonyl)-6-methyl-2H-chromen-2-one* (Entry 5, Table 5): The reaction of 2-hydroxy-5-methylbenzaldehyde (0.163 g, 1.2 mmol), dimethyl malonate (0.158 g, 1.2 mmol) and L-proline (0.156 g, 1.2 mmol) in undried [MMIm][MSO_4_] (2 mL) afforded the corresponding product; yield: 0.220 g (87%); white crystals (EtOH); mp 127–128 °C; ^1^H-NMR (400 MHz, CDCl_3_, ppm) δ 8.53 (s, 1H), 7.47 (dd, *J* = 1.8, 8.1 Hz, 1H), 7.40 (s, 1H), 7.28 (d, *J* = 8.1 Hz, 1H), 3.97 (s, 3H), 2.44 (s, 3H); ^13^C-NMR (100.6 MHz, CDCl_3_, ppm) δ 163.8, 157.0, 153.4, 149.2, 135.7, 134.8, 129.2, 117.7, 117.6, 116.5, 52.9, 20.7; ν_max_(NaCl)/cm^−1^ 3034, 2942, 2844, 1755; Electrospray MS (micrOTOF Focus) *m/z* (%) 241 [M + Na^+^] (52), 220 [M + H^+^ + 1] (13), 219.06575 [M + H^+^] (C_12_H_11_O_4_ requires 219.06519, 100), 203 (30), 189 (18), 171 (17).

*3-(Methoxycarbonyl)-8-methyl-2H-chromen-2-one* (Entry 7, Table 5): The reaction of 2-hydroxy-3-methyl-benzaldehyde (0.163 g, 1.2 mmol), dimethyl malonate (0.158 g, 1.2 mmol) and L-proline (0.156 g, 1.2 mmol) in undried [MMIm][MSO_4_] afforded the corresponding product; yield: 0.245 g (94%); yellow needles (EtOH); mp 106–107 °C; ^1^H-NMR (400 MHz, CDCl_3_, ppm) δ 8.56 (s, 1H), 7.51 (d, *J* = 7.5 Hz, 1H), 7.45 (d, *J* = 7.8 Hz, 1H), 7.24 (t, *J* = 7.7 Hz, 1H), 3.97 (s, 3H), 2.48 (s, 3H); ^13^C-NMR (100.6 MHz, CDCl_3_, ppm): 163.9, 156.9, 153.6, 149.6, 135.7, 127.2, 126.4, 124.4, 117.6, 117.4, 52.9, 15.4; ν_max_(NaCl)/cm^−1^ 3051, 2953, 2847, 1761; Electrospray MS (micrOTOF Focus) *m/z* (%) 257 [M + K^+^] (7), 242 [M + Na^+^ + 1] (8), 241 [M + Na^+^] (65), 220 [M + H^+^ + 1] (17), 219.06524 [M + H^+^] (C_12_H_11_O_4_ requires 219.06519, 100), 201 (9), 187 (29), 175 (4).

*6-Bromo-8-methoxy-3-(methoxycarbonyl)-2H-chromen-2-one* (Entry 11, Table 5): The reaction of 5-bromo-2-hydroxy-3-methoxybenzaldehyde (0.231 g, 1 mmol), dimethyl malonate (0.132 g, 1 mmol) and L-proline (0.130 g, 1 mmol) in undried [MMIm][MSO_4_] (2 mL) afforded the corresponding product; yield: 0.310 g (99%); white powder (EtOH); mp 209–210 °C; ^1^H-NMR (400 MHz, CDCl_3_, ppm) δ 8.45 (s, 1H), 7.32 (d, *J* = 1.9 Hz, 1H), 7.26 (d, *J* = 1.8 Hz, 1H), 3.98 (s, 3H), 3.96 (s, 3H); ^13^C-NMR (100.6 MHz, CDCl_3_, ppm) δ 163.4, 155.5, 148.0, 147.7, 144.0, 122.5, 119.3, 119.2, 118.8, 117.1, 56.6, 53.1; ν_max_(NaCl)/cm^−1^ 3080, 2965, 1761; Electrospray MS (micrOTOF Focus) *m/z* (%) 337 [M + Na^+^ + 2] (59), 335 [M + Na^+^] (60), 315 [M + H^+^ + 2] (43), 312.97004 [M + H^+^] (C_12_H_10_BrO_5_ requires 312.97061, 38), 259 (17), 233 (11), 203 (100), 189 (84), 175 (50), 171 (26), 159 (15), 157 (9).

*3-(Methylsulfonyl)-2H-chromen-2-one* (Entry 13, Table 5): The reaction of salicylaldehyde (0.183 g, 1.5 mmol), ethyl methylsulfonyl acetate (0.166 g, 1 mmol) and L-proline (0.130 g, 1 mmol) in undried [MMIm][MSO_4_] (2 mL) afforded the corresponding product; yield: 0.3214 g (96%); white needles (EtOH); mp 184–185 °C; ^1^H-NMR (400 MHz, CDCl_3_, ppm) δ 8.67 (s, 1H), 7.76 (m, 2H), 7.45 (t, *J* = 8.1 Hz, 2H), 3.36 (s, 3H); ^13^C-NMR (100.6 MHz, CDCl_3_, ppm) δ 156.0, 155.3, 147.6, 135.5, 130.4, 127.6, 125.6, 117.2, 117.1, 41.7; ν_max_(NaCl)/cm^−1^ 3052, 2933, 1743; Electrospray MS (micrOTOF Focus) *m/z* (%) 248 [M + Na^+^ + 1] (14), 247 [M + Na^+^] (87), 226 [M + H^+^ + 1] (10), 225.02098 [M + H^+^] (C_12_H_9_O_4_S requires 225.02161, 100).

*7-Hydroxy-3-(methylsulfonyl)-2H-chromen-2-one* (Entry 14, Table 5): The reaction of 2,4-hydroxy-benzaldehyde (0.154 g, 1.1 mmol), ethyl methylsulfonyl acetate (0.182 g, 1.1 mmol) and L-proline (0.142 g, 1.1 mmol) in undried [MMIm][MSO_4_] (2 mL) afforded the corresponding product; yield: 0.260 g (99%); yellow needles (EtOH); mp 295–296 °C; ^1^H-NMR (400 MHz, MeOD, ppm) δ 8.65 (s, 1H), 7.73 (d, *J* = 8.6 Hz, 1H), 6.91 (dd, *J* = 2.2, 8.6 Hz, 1H), 6.80 (d, *J* = 2.2 Hz, 1H), 3.29 (s, 3H); ^13^C-NMR (100.6 MHz, MeOD, ppm) δ 166.8, 159.3, 158.4, 149.4, 133.7, 123.4, 115.9, 111.6, 103.5, 42.0; ν_max_(NaCl)/cm^−1^ 3297, 3062, 2933, 1712; Electrospray MS (micrOTOF Focus) *m/z* (%) 263 (M + Na^+^) (100), 241.01573 (M + H^+^), (C_10_H_9_O_4_S requires 241.01652, 80), 225 (22), 201 (60).

*7-Bromo-3-(methylsulfonyl)-2H-chromen-2-one* (Entry 15, Table 5): The reaction of 4-bromo-2-hydroxy-benzaldehyde (0.151 g, 0.75 mmol), ethyl methylsulfonyl acetate (0.083 g, 0.5 mmol) and L-proline (0.065 g, 0.5 mmol) in undried [MMIm][MSO_4_] (2 mL) afforded the corresponding product; isolated yield: 0.141 g (93%); yellow powder (EtOH); mp 254–255 °C; ^1^H-NMR (400 MHz, CDCl_3_, ppm) δ 8.61 (s, 1H), 7.65 (s, 1H), 7.58 (s, 2H), 3.35 (s, 3H); ^13^C-NMR (100.6 MHz, CDCl_3_, ppm) δ 155.3, 155.2, 146.8, 131.0, 130.3, 129.3, 127.7, 120.5, 115.9, 41.7; ν_max_(NaCl)/cm^−1^ 3066, 2927, 1749; Electrospray MS (micrOTOF Focus) *m/z* (%) 327 (M + Na^+^ + 2) (84), 325 (M + Na^+^) (64), 305 (M + H^+^ + 2) (88), 302.93216 (C_10_H_8_O_4_SBr requires 302.93212) (100), 201 (56).

## 4. Conclusions

1,3-Dimethylimidazolium methyl sulfate, [MMIm][MSO_4_], an inexpensive, easily prepared, low-viscosity ionic liquid with low cytotoxicity, can act efficiently as the solvent and catalyst of Knoevenagel condensation reactions between 4-substituted benzaldehydes and active methylene compounds without any promoter other than a small amount of water (≈2%) such as can be introduced simply by exposure to air; these reactions are clean and are completed at room temperature within a few minutes, work-up is simple, and the reaction medium can be reused several times without significant reduction in yield. When used to cyclize *o*-hydroxybenzaldehydes with active methylene compounds to obtain coumarins it was necessary to use L-proline as promoter: Heating the reagents and promoter at 90 °C in undried [MMIm][MSO_4_] afforded high yields of the desired coumarins. Some of the coumarins are described for the first time.
